# Case of Continued Fever, Attended with Some Truly Anomalous Appearances

**Published:** 1818-05

**Authors:** John Syer


*For the London Medical and Physical Journal.*

### *Case of continued Fever, attended with some truly anomalous Appearances.* By JOHN SYER, Esq.

ON Wednesday, March 4th, I was directed to visit a young woman, 17 years of age, extremely ill with fever, accompanied with delirium. The patient had twice before, at remote periods, been afflicted with attacks of continued fever. The early history of this is as follows:—On Monday, 24th February, she found herself incapable of pursuing her work, as a servant, and continued very unwell until Wednesday, when she experienced a most severe rigor, with violent head-ach and delirium the same night. Her mother, on the same evening, administered an emetic, as she complained of a good deal of sickness: it operated powerfully, and she voided a good deal of bile. The delirium, though unattended with any violence, was seldom absent, day or night, but always returned with the evening paroxysm in an increased degree, depriving her almost entirely of sleep. Her friends, during an interval of a week, had sought no medical advice; perhaps judging, from the past experience of her recoveries, though even then the disease was severe, that it would run its course with impunity, with the help of very little medicine. On Sunday, she was covered with a papulous eruption, of a scarlet appearance, resembling measles, but rather more elevated; the skin, in the intermediate spaces, having no particular efflorescence. The fever and delirium continued as before; her bowels were pretty regular; her urine high coloured, but deposited early, and continued so to do, a copious lateritious sediment. Her parents still did not think it necessary to call in advice, until Tuesday, 3d of the present month, when a friend, united with me in practice, thought, at the first glance, that the disease was Scarlatina; and directed a purgative, with saline and antimonial medicines.

On Wednesday, 4th March, I visited her, and found, to my astonishment, the patient sitting up; for which I rebuked the attendants. Finding a pulse almost imperceptible, a good deal of nausea, and a cold clammy skin, I enjoined constant confinement in bed. Her tongue, except merely at the edges, was covered with a dry white crust, which, according to the report of her mother, had been its condition from the beginning in a very marked degree. She was thirsty, and the pulsation of the artery at the wrist, as well as the temple, so indistinct, that I could scarcely perceive them. She was directed to have a blister *inter scapulas*, and to take a common saline mixture, with Sper. Ætheris

Sulphuric. and the Pulv. Contrayervæ, &c. with half a grain of antimonial powder, every four or five hours.

On Thursday, 5th March, I found she had no sleep the preceding night; that her delirium was equally strong, and with much restlessness; there was some *subsultus tendinum*, and the tongue had a slight brownish fur in the centre. Upon enquiry as to the state of the head and of vision, she frequently complained of pain in the forehead; and her sight was so imperfect that she could not discern the smallest print.

Three days ago, the catamenia appeared, rather profusely, although she had been affected in a similar way a fortnight before. The eruption was still universal and elevated, more prominent about the arms and legs than about the shoulders and breast. I directed the following medicine: —℞. Confect. Aromat. ʒifs. Spir. Æther. Sulph. ʒiij. Spir. Æth. Nitros. ʒij. Tr. Opii. gtt. xx. Mist. Salin commun. ℔ fs. Sumat. Ægra Cochlear. tria ampla 5tis. horis. It may be observed of the delirium, that it has not been hitherto marked by any degree of vigorous action in the arterial system. At night, a pill was ordered, of opium and pulv. ipecacuanha. The bowels were sufficiently open; but the eruption about the shoulders and neck was of an ugly purplish hue. She sighs frequently, and seems uneasy about the chest; complains of universal soreness of the integuments, especially about the eyes and forehead. The urine is high coloured, without sediment for the last two days. The appearances of the eruption evidently vary with the temperature of the skin, which has seldom equalled that of regular continued fever.

March 5.—The opiate produced some tranquil sleep; its effects continued during the day; but, when awake, she is generally moaning and uneasy. She is rather more flushed in the face, and has complained of pain in the head: pulse still obscurely felt. She labours under a troublesome cough, and expectorates a thick mucus. The eruption still very vivid in every part of the body, except the shoulders.

March 6.—She passed a better night, but there appears to be rather more determination to the head: the opiate omitted last night. The eruption very confluent in the face, and florid in other parts. The eyes very vascular, and she is less rational. The bowels not having been relieved for the last thirty-six hours, she was directed to take four grains of the submuriate of mercury, with eight of socotorine aloes; and, if her head was not much relieved of pain in the evening, a dozen leeches to the forehead. The tongue is now extremely dry, with a copious, thick, whitish crust; but there is an appearance of sordes about the gums, the frequent precursory sign of low symptoms. There is still *subsultus tendinum*, but not quite so strong. Her constant cry has been for cold water; and she has, till the present time, been prevailed on to take but little besides. ℞. Confect. Arom. ʒij. Tr. Cascarillæ, ʒi. Tr. Opii. gtt. xij. Aq. puræ, ℥vi. sumatur ut antea post alvi solutionem. The fever this evening was much less severe than at noon. I had a better opportunity of feeling the pulse, which was not very weak, and about 120. The tongue moister at the edges, and the fur a little detached. She gave rather more rational answers, but complained of so much weakness that she could not turn in bed. Complained less of her head. Has taken rather more nourishment. Urine clear, and not high coloured. The eruption about the neck and shoulders put on more of a petechial character. Wine and broth were administered as often as the patient could be prevailed upon to take them. It was to be regretted that the circumstances of her friends would not admit of that free exhibition of wine which the case seemed to require.

March 7.—The patient was much better this morning. The eruption was still visible about the shoulders and body, but less conspicuous about the arms, though it is still elevated on the legs. Throat slightly sore, and cough frequently violent. Urine clear; tongue still moist at the edges, has less fur, and that slightly brown. She is more collected in her intellects, and has less pain: indeed, she seemed quite free from it to-day at noon. The pulse is more feeble; considerable trembling, and some *subsultus tendinum* are still observable. She had two healthy bilious evacuations since yesterday, and slept soundly for an hour and a half in the night. Repetatr. mistura ut heri. In the evening, still continued, on the whole, better. The delirium is less constant; the eruption is gradually retreating, except about the neck and shoulders; tongue cleaner, and she takes nourishment more freely; yet her pulse is very feeble, quick, and rather intermittent.

March 8, (which, from the commencement of the disease, was the thirteenth day.)—I have observed hitherto no appearance of crisis, except in the casual appearances of the urine, and these have been very uncertain. The skin is perfectly dry and hard, and there is very little preternatural heat at any time. She is not so well to-day; yet the delirium is much on the decline, and she gets more tranquil sleep in the night. Upon the whole, she is rather comatose, and makes incoherent answers, unless perfectly awakened. There is much less eruption, and it is every where level with the skin, attended with partial disquamation. The urine is pale, highly turbid, of a milky cast, and deposits no precipitation. Bowels quite regular; tongue rather more natural, with very little fur, of a whitish aspect. The pulse is now palpable to the touch, not quite so intermittent, and about 120. She takes milk and good broth, with wine occasionally. In the evening, she complained more of debility; *subsultus tendinum* remains. Has had very frequent and deep sighs for the last three or four days, according to the report of her attendants; and sometimes I have noticed a spasmodic affection of the breath, which has occasionally alarmed her, and may serve to account, in some measure, for the irregularity of her pulse.

The following mixture was directed:—℞. Pulv. Cinchonæ flav. ʒiij. Spir. Æth. Sulph. ʒiiſs. Tr. Gent. comp. ℥ſs. Spir. Ammon. comp. ʒij. Aq. puræ, ℥vij. f. mist.—sumat Cochl. iij. ampla 4ta. vel. 5ta. quaque horâ. Wine and broth continued as usual.

March 9.—In the forenoon, she appeared better, and was more rational than heretofore; the tongue was tolerably clean and moist; however, the stupor was rather increased. Pulse 130, but not intermittent; and she passed the preceding night favourably.—Evening—The symptoms became worse in every respect.

March 10.—Her tongue was now exceedingly brown in the centre, and lined with a thick white crust for a considerable space about the edges. The urine was very dark coloured when recently voided, but deposited a very singular dirty-brown sediment. She was much more comatose, and was mostly picking the bed-clothes. The *subsultus tendinum* much increased; the eruption has quite disappeared. There is now more difficulty in administering both food and medicine, which she takes in very small quantities at a time. The eyes appeared natural, and the patient's sight is very correct. Pulse 128, but regular, and very feeble. Her bowels were yesterday relieved by opening medicine. I directed a blister to each leg internally, and a table-spoonfull of yeast once in six hours, with as much wine as could be given.—In the evening, she was still more comatose, and it was with the utmost difficulty she could be roused; pulse becoming weaker, and occasionally intermittent. For the last three days, we have observed a slight rash, with a good deal of efflorescence in the face. The symptoms were, in other respects, as in the morning.

March 11.—She is now highly insensible and comatose. Pulse the same in frequency, but weaker; tongue universally dry and brown; makes no complaint whatever, but is generally picking the clothes. *Subsultus tendinum* much increased; the urine has the same dirty appearance. She takes scarcely any thing in the way of food or medicine, but in the smallest quantities. The cough has ceased ever since the comatose symptoms appeared.

March 12.—The patient evidently becomes weaker: her situation is truly critical; sometimes she is very refractory, and unwilling to be roused. Tongue much the same; pulse excessively feeble, and rather irregular; has voided no urine since yesterday afternoon, but there is no fullness or sense of narrowness about the hypogastric region. She was directed to take a cup of senna tea, with a view of relieving the intestines, and of exciting a discharge of urine. Sinapisms were ordered to the soles of the feet yesterday, but, through the negligence of the attendants, were not applied.

March 13.—Yesterday evening, I took the report of a friend, who found her much worse in every respect, and observed that she was a good deal convulsed, and could scarcely swallow any thing; and this morning, about three o'clock, she expired.

The symptoms of the preceding case, for some days previous to the patient's death, resembled those of typhus, which was not very unlikely to occur, considering that this disease was rather endemic in the town, and that all acute disorders are apt to partake of the character of the prevailing endemic or epidemic of the season. I forbear to offer any further remarks; but should feel a pleasure in receiving any comments from your pen, or from those of your numerous correspondents.

*Atherstone, Warwickshire; March* 15, 1818.

*** As there is an epidemic in his neighbourhood, we shall be thankful for other communications from this candid correspondent. If we might offer an opinion at this distance, we should advise more free evacuations, especially in the early stage of such fevers; and never press wine or nourishment against the inclination of the patient, but indulge freely the wish for water.

